# Survival Status and Predictors of Mortality among Multidrug-Resistant Tuberculosis Patients in Saint Peter's Specialized Hospital, Addis Ababa, Ethiopia

**DOI:** 10.1155/2021/6696199

**Published:** 2021-09-03

**Authors:** Mikyas Arega Muluneh, Abayneh Birlie Zeru, Behailu Tariku Derseh, Abebaw Molla Kebede

**Affiliations:** ^1^Department of Midwifery, Institute of Medicine and Health Sciences, Debre Berhan University, Debre Berhan, Ethiopia; ^2^Department of Public Health, Institute of Medicine and Health Sciences, Debre Berhan University, Debre Berhan, Ethiopia; ^3^Department of Nutrition and Reproductive Health, School of Public Health, Mizan-Tepi University, Tepi, Ethiopia

## Abstract

**Background:**

Multidrug-Resistant Tuberculosis (MDR-TB) is tuberculosis that is resistant to at least both rifampicin and isoniazid. The World Health Organization as reported in 2019 revealed that Ethiopia is among the 20 countries with the highest estimated numbers of incident MDR-TB cases. However, supporting evidence is limited in the study area after the Ethiopian national strategic plan for tuberculosis prevention and control is started.

**Objective:**

To determine survival status and predictors of mortality among multidrug-resistant tuberculosis patients treated in Saint Peter's Specialized Hospital at Addis Ababa, Ethiopia, 2020.

**Methods:**

An institutional retrospective cohort study was conducted using all MDR-TB patients who were enrolled in Saint. Peter's Specialized Hospital from January 01, 2015, to December 31, 2017. A pretested data extraction form that had 5 items for sociodemographic and 15 items for the measurement of clinical characteristics of 484 MDR-TB patients was used. STATA software version 14.2 was used for data cleaning and analysis. A variable that fitted in the bivariable Cox proportional hazard model at *p* value <0.25 was used in the final multivariable Cox proportional hazard model, and independent predictors of time to event were determined at a *p* value of 0.05.

**Result:**

A total of 484 patients were followed up for 5,078 person-months. Among the total patients, nearly half, 238 (48.8%), were males. The median age of patients was 30 years (interquartile range (IQR), 24–39), and 56 (11.6%) were aged between 1 and 19 years. During the follow-up period, 315 (65.1%) patients were cured, 125 (25.8%) completed treatment, 24 (5%) died, and 20 (4.1%) were lost to follow-up. The overall cumulative probability survival of the patients at the end of treatment was 94.85% (95% confidence interval (CI): 92.38%–96.53%). The independent predictors of time to death were being anemic (AHR = 3.65; 95% CI: 1.36, 9.79), having clinical complication (AHR = 3; 95% CI: 1.2, 7.5), and being HIV infected (AHR = 5.8; 95% CI: 2.2, 15.7).

**Conclusions:**

MDR-TB patients' survival rate was high in St Peter's Specialized Hospital. MDR-TB patients with anemia, HIV coinfection, and clinical complications had higher risk of mortality. So, prevention and controlling of anemia, HIV/AIDS, and clinical complications will reduce the mortality of MDR-TB patients.

## 1. Introduction

Multidrug-resistant tuberculosis (MDR-TB) is tuberculosis (TB) disease resistant to at least both rifampicin and isoniazid, the two most powerful anti-TB drugs. Detection of MDR-TB requires bacteriological confirmation of TB and testing for drug resistance using rapid molecular tests, culture methods, or sequencing technologies. Treatment requires a course of second-line drugs at least for 9 months and up to 20 months, supported by counseling and monitoring for adverse outcomes [1, 2].

TB remains a major cause of ill health and is one of the top 10 causes of death worldwide. An estimated 10.0 million people fell ill with TB in 2018. Globally, there were 1.2 million TB deaths among HIV-negative people in 2018 and an additional 251000 deaths among HIV-positive people. Since 2007, TB has been the leading cause of death from a single infectious agent, ranking above HIV/AIDS. In 2018, there were an estimated 390,000 new cases of multidrug-resistant TB [1].

An anti-TB drug resistance surveillance data estimated 4.1% of new and 19% of previously treated TB cases in the world to have either multidrug-resistant or rifampicin-resistant tuberculosis (MDR/RR-TB). In 2016, an estimated 600,000 new cases of MDR/RR-TB emerged globally and caused 240,000 deaths. About 6.2% of MDR-TB cases have additional drug-resistance, extensively drug-resistant TB (XDR-TB) [3].

World Health Organization reports in 2019 reveal that Ethiopia is among the 20 countries with the highest estimated numbers of incident MDR-TB cases and listed among 14 countries that are the highest for TB, TB/HIV, and MDR-TB cases [1]. A major barrier to progressing toward TB elimination in Ethiopia is the TB “case detection rate” of only 60 percent, meaning that an estimated 80,000 Ethiopians who developed TB in 2014 were never diagnosed or treated, leading to ongoing spread of TB to family members and communities. The gap in case detection rate is even worse for the more severe MDR-TB, where less than a quarter of an estimated 2,200 Ethiopian MDR-TB patients are identified each year [4].

Different factors have been associated with mortality of MDR-TB patients. Some studies show that clinical complication, the presence of any chronic disease including HIV [5–7], older age [7, 8], therapeutic delay [5, 7], smoking [5], body mass index (BMI) less than 18 [9], poor drug adherence [6], alcohol use [7], hypokalemia [8], low CD4 [9], extra-pulmonary TB [6], and living in rural areas [8] are risk factors for the mortality of MDR-TB patients. However, contradictory evidence revealed that BMI, HIV status, extra-pulmonary TB [8], and smoking [7] are not risk factors for mortality of MDR-TB patients. So, the purpose of this study was to assess survival status and identify predictors of mortality among MDR-TB patients in Saint Peter's Specialized Hospital, Addis Ababa, Ethiopia.

## 2. Methods and Materials

### 2.1. Study Design and Period

An institution-based retrospective cohort study design was used to assess the survival status and predictors of mortality among MDR-TB patients treated in Saint Peter's Specialized Hospital, Addis Ababa, Ethiopia, in March, 2020.

### 2.2. Study Setting and Population

The study was conducted in Addis Ababa, the capital city of Ethiopia. It is the largest city in Ethiopia, established in 1887 by emperor Menilik II. It has the status of both a city and a state. The city is divided into ten subcities. In 2017, it had a projected 3,435,028 million population of whom 1,809,577 were females and the rest 1,625,451 were males [10].

The study was conducted in Saint Peter's Specialized Hospital, the largest TB referral center in the country, in Addis Ababa. The hospital has a long history of TB management since it is a hospital where TB case management was initiated for the first time in the country in June 1961. The study population was MDR-TB patients who started MDR-TB treatment from January 1, 2015, to December 31, 2017, and then, by considering the lately registered patients, we followed them up to the end of their treatment follow-up based on records which take up to December 2019. Five hundred and nineteen MDR-TB patients were managed at inpatient and outpatient levels during the indexed period.

All completely registered documents that contain identified variables of treated patients at Saint Peter's Specialized Hospital during the index period were included in the study. All transfer-out patients' documents whose treatment outcome was not available were excluded. By excluding 28 transfer outpatients and 7 patients with incomplete medical records, all the remaining 484 MDR-TB patients managed form 2015–2017 were included in the study.

### 2.3. Data Collection Tool and Procedures

A structured data extraction form was prepared in English language by reviewing previous similar works of literature and considering the availability of variables on medical registration books and treatment cards [5, 8, 11–13]. There were 5 items for sociodemographic and 15 items for the measurement of clinical characteristics of MDR-TB patients. The baseline weight of patients was taken as recorded on the patient card nearest to 0.5 kilograms. Baseline weight was categorized using the median weight of patients. Nothing was written about how the weight was measured. The hemoglobin level was recorded to the nearest 0.1 g/dl. The CD4 count of HIV/AIDS patients was measured in number of cells per cubic millimeter. The person-time of patients was measured from the time of diagnosis of MDR-TB to event (death). A pretest was conducted on records of 25 MDR-TB patients that started treatment in 2014 at St. Peter's Specialized Hospital. Then, drug adherence and height variables were excluded because more than half of the records missed these variables.

Data were extracted by one BSc nurse who has been working at St. Peter's Specialized Hospital and 2 general practitioners (GPs), new graduates from Debre Berhan University.

### 2.4. Operational Definition

- Death (event): a patient who died of any cause during the course of MDR-TB treatment [14]
- Censored: when the outcome of interest has not been observed for an individual; this includes cured completed, treatment failure, and defaulter [15]
- Cured: treatment completed according to national recommendation without evidence of failure and three or more consecutive cultures taken at least 30 days apart are negative after the intensive phase [14]
- Treatment completed: treatment completed according to national recommendation without evidence of failure but no record that three or more consecutive cultures taken at least 30 days apart are negative after the intensive phase [14]
- Treatment success: sum of cured and completed treatment [14]
- Treatment failure: treatment terminated or need for permanent regimen change of at least two anti-TB drugs at the end of the intensive phase or later during treatment [14]
- Defaulter (lost follow-up): a patient whose treatment was interrupted for two consecutive months or more for any reason without medical approval [14, 16]
- Time to death: time to occurrence of death measured from confirmed diagnosis of MDR-TB to event (death) (40)
- Therapeutic delay: a confirmed MDR-TB patient who starts treatment after one month [5]
- Previously NOT treated for TB: patients have never been treated for TB or have taken anti-TB drugs for less than a month [14]
- Previously treated case: a patient who admits having been treated for TB for one month or more [14]
- Clinical complications: disorders that occur during the course of MDR-TB treatment
- Anemia: <13 g/dl for adult men, <11 g/dl for age 6 months to 5 years, and <12 g/dl for nonpregnant women and children aged 6–14 years were applied [17]

### 2.5. Data Quality Control

Orientation was given for half a day on the objectives and relevance of the study, confidentiality of information, and how to extract data on MDR-TB logbook and treatment cards. Daily supervision of the data collection process and checking the completeness and consistency collected data were carried out by the principal investigator.

### 2.6. Data Processing and Analysis

First, data were checked for completeness and consistency before entering into computer. Then, data were coded, entered into Epi-Data version 3.1 Software, and exported to STATA statistical software version 14.2 for data cleaning and analysis.

A Shapiro–Wilk test (*p* value <0.05) showed that age, baseline weight, hemoglobin, and CD4 counts were not normally distributed. The descriptive analysis such as frequency distribution, percentages, and measures of central tendency for continuous variables (median and interquartile range (IQR)) was used. The maximum variance inflation factor was 1.26 among variables in the final model. A Kaplan–Meier Survival curves were carried out to compare survival probability between groups using a log-rank test. The incidence of death with respect to person-time at risk was calculated. A variable that fitted in the bivariable Cox proportional hazard model at *p* value <0.25 was used in the final multivariable Cox proportional hazard model, and independent predictors of time to event were determined at a *p* value of <0.05.

The value of Harrell's C was 0.9638 for the assessment of model adequacy. The proportionality assumptions to the Cox proportional hazard model were checked using the goodness-of-fit (GOF) test by Schoenfeld residual. All candidate covariates for multivariable Cox regression did not violate the proportionality assumptions.

The goodness of fit of the final model was checked by likelihood ratio. Results of the likelihood ratio test were a chi-square of 104.47 with a *p* value of <0.0001 suggesting that the model was good fitted.

Crude and adjusted hazard ratios with 95% CI were calculated to measure the magnitude of the association between the covariates and time to MDR-TB death. A *p* value of <0.05 was taken as a cutoff point to identify statistically significant variables.

## 3. Result

### 3.1. Sociodemographic Profile

The median age of patients was 30 years (IQR, 24–39), and 56 (11.6%) were aged between 1 and 19 years. Among the total patients, over half of the patients, 248 (51.2%), were females. A majority, 401 (82.9%), were urban dwellers. More than one-third of patients, 185 (38.2%), were government employees followed by 98 (20.2%) students.

About three-fifths of the patients (297, 61.4%) and a quarter (120, 24.8%) were from Addis Ababa and Oromia region, respectively (Table 1).

### 3.2. Clinical Characteristics and Comorbidities

Near to three-fourth, 359 (74.5%), of the patients were diagnosed through Gene Xpert followed by 72 (14.9%) culture, 46 (9.5%) line probe assay, and 7 (1.4%) clinical method. More than three-fourth, 389 (80.4%), of the MDR-TB patients had pulmonary TB. Almost one-third, 155 (32.0%), of the MDR-TB patients were new cases without previous history of anti-TB medication. At the commencement of treatment, the median baseline weight was 45.5 kg (IQR: 40–52 kg) and 172 (35.5%) had less than 43 kg. Regarding the history of substance use, three-fourths, 363 (75.0%), were nonusers, 105 (21.7%) were alcohol users, and 14 (2.9%) were khat chewers.

A majority (392, 81.0%) of the patients started treatment on the same day of a confirmed diagnosis, 91 (18.8%) within 10 days, and 1 (0.2%) after 30 days of confirmed MDR-TB diagnosis. Almost all MDR-TB patients 463 (95.7) developed adverse drug reactions during the course of treatment. Of 484 MDR-TB patients, 96 (19.8%) had at least one clinical complication throughout the course of treatment. The leading complication was pneumonia 59 (12.2%) followed by hemoptysis 16 (3.3%).

As shown in Table 2, all MDR-TB patients had been tested for HIV and 96 (19.8%) were HIV positive. The median CD4 count of HIV-infected patients was 165 cells/mm^3^ with a range of 46–800 cells/mm^3^. All HIV coinfected patients were on first-line ART drug regimen; 92 (96.8%) were taking tenofovir disoproxil fumarate-lamivudine-efavirenz (TDF-3TC-EFV), and 3 (3.2%) were on azidothymidine-lamivudine-efavirenz (AZT-3TC-EFV) drug regimen. One HIV-positive MDR-TB patient was not started ART at the time of diagnosis.

The median baseline hemoglobin level was 13.4 g/dl (IQR: 12.2–14.5 g/dL), and more than a quarter of patients 133 (27.5%) were anemic at the time of MDR-TB diagnosis.

Fifty-one (10.1%) patients had chronic diseases other than HIV and anemia. Among other chronic diseases, 19 (3.7%) had diabetes and 18 (3.7%) had cardiac illness (Table 2).

### 3.3. Treatment Outcome

Of the 484 MDR-TB patients, 315 (65.1%) were cured and 125 (25.8%) completed treatment that makes the proportion of MDR-TB patients who successfully completed treatment 440 (90.1%), 24 (5%) dead, and 20 (4.1%) lost follow-up (Table 3).

### 3.4. Survival Time

All 484 patients were followed up for a median of 247.5 days (IQR: 237 to 497.75 months) ranging from 2 days to 1340 days. The total person-days of follow-up was 158,337 days. During this period, 24 deaths were recorded, resulting in 1.5 deaths per 10,000 person-days. The median time to death was 57.5 days (IQR, 30–95 days), indicating 75% of deaths occurred during the first 95 days of the MDR-TB course of treatment.

**Figure 1:**
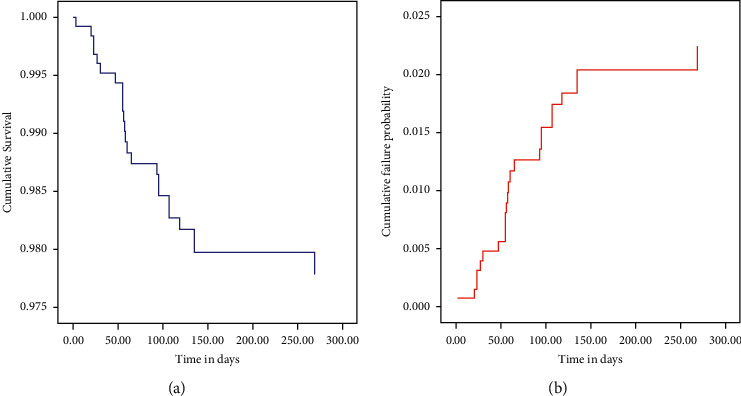
(a) Cumulative survival estimate and (b) cumulative failure probability of MDR-TB patients in St. Peter's Specialized Hospital, 2020.

**Figure 2:**
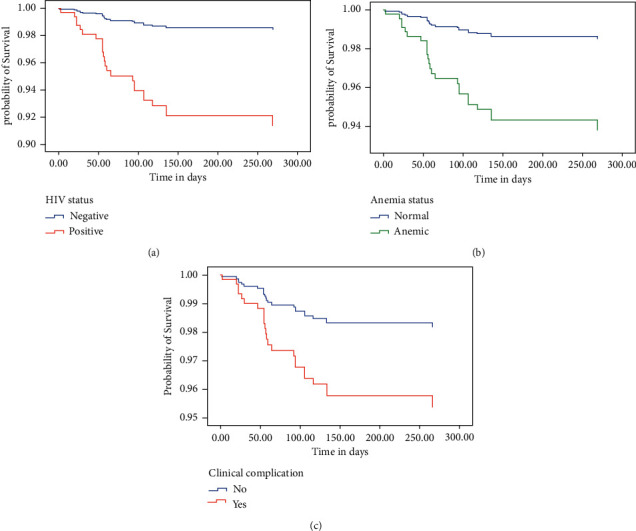
Covariate adjusted survival function of MDR-TB patients in SPSH, 2020. (a) Stratified by HIV status, (b) stratified by anemia status, and (c) stratified by the presence of clinical complication.

The cumulative survival rate of MDR-TB patients at the end of treatment was 94.9%. At six months of the MDT-TB course of treatment, the survival probability was 95.2%. The cumulative failure probability of patients was high in the first six months of follow-up (Table 4 and Figures 1 and 2).

### 3.5. Factors Associated with MDR-TB Death

As shown in Table 5, with bivariable analysis, baseline weight, hemoglobin level, HIV status, previous history of treatment, clinical complication, and the presence of lung cavities were significantly associated with the time of death. However, in multivariable binary Cox regression, being anemic, HIV coinfection, and clinical complications were significantly associated with death during MDR-TB medication. Those who had clinical complications were more than 3 times at risk to have a death outcome than those who had no clinical complications (AHR = 2.55; 95% CI: 1.06, 6.10). HIV-infected patients were about 6-folds at risk to have a death outcome than noninfected MDR-TB patients (AHR = 5.7; 95% CI: 2.2, 14.51), and anemic patients were more than 4-folds at risk than nonanemic patients (AHR = 4.26; 95% CI: 1.65, 11.02) (Table 5).

## 4. Discussion

A retrospective cohort study was conducted among 484 MDR-TB patients in Saint Peter's Specialized Hospital, Addis Ababa, Ethiopia, to assess the survival status and predictors of mortality. A majority, 440 (90.9%) (65.1% cured and 25.8% completed), of the patients had successful treatment outcomes, which reflects Saint Peter's specialized Hospital has achieved the “Global Plan to END TB 2016–2020” target of ≥87% [18].

The survival rate of 94.85% of MDR-TB patients in St. Peter's Specialized Hospital has shown considerable improvement compared to the 78.95% report in the same hospital 7 years ago [6]. It is also higher than 88% in SNNPR [14] and 80% in Wuhan, China [16].The difference might be due to the change in time that is related to increased patients' awareness of their health and improvement in patient care.

The death rate was 1.5 per 10,000 person-days which is lower than the report of Wuhan, China, which was 1.79 per 10,000 person-days, SNNPR, Ethiopia, 1.92, and much lower than the incidence rate in this hospital before 7 years which was 4 per 10,000 person-days [5, 12, 19]. The possible reason for these findings is as follows: first, it might be because the setting is a TB-specialized hospital that has good infrastructure with highly trained and skilled healthcare workers and the site is a research center for tuberculosis that helps the hospital to identify gaps to intervene for good achievement. Second, chronic diseases get more emphasis that decreases the mortality of patients related to it.

Almost all deaths of patients, 23 (95.8%), occurred in the first 6 months of the MDR-TB treatment course which is consistent with a study report in Northwest Ethiopia [11]. The following would be the possible explanations: Firstly, a diagnosis of MDR-TB and being hospitalized for 6 months might create psychosocial problems that led to a poor prognosis. Secondly, more than one-third were from out of Addis Ababa, so the distance might have led to feeling like a stranger and it would be difficult to trace defaulters. Lastly, the majority (66.7%) of the patients who died were on ART, and taking many drugs might have affected their psychological wellness that led to poor prognosis.

Hemoglobin level was one of the factors associated with mortality of MDR-TB patients in this study. Anemic patients were more than 4-folds at risk of death during MDR-TB medication than nonanemic patients. This result is consistent with a study from Northwestern Ethiopia, Tanzania, and Haiti that describes anemia was an associated factor to the death of patients [11, 20, 21].

Another factor associated with death of patients was HIV/AIDS coinfection. HIV-infected patients were 5.7 times more at risk to die while on MDR-TB treatment. This is in line with findings in the Amhara Region of Ethiopia, SNNPR, Ethiopia, South Africa, and Latvia [5, 8, 12, 22, 23]. HIV/AIDS might compromise their immunity and lead to poor prognosis of their health status.

Those who had clinical complications were 2.55 times at risk of death than those who had no clinical complications. Other studies have also shown that MDR-TB patients with clinical complications were associated with an increased risk of death [5, 19]. Clinical complications may adversely affect the prognosis of patients involving the worsening of MDR-TB and affect other organ systems.

This study had limitations of inability to control potentially important confounding variables such as drug adherence, economic status, nutritional status, and educational factors which were not assessed to determine their relationship with the death of MDR-TB patients.

## 5. Conclusions

The probability of survival during MDR-TB medication was high that indicates St. Peter's Specialized Hospital has achieved 2020 milestones for a reduction in tuberculosis-related deaths planed by the World Health Organization. Almost all deaths of MDR-TB patients were during the first six months of treatment. Being anemic, HIV coinfected cases, and clinical complications were associated with mortality from MDR-TB.

## Figures and Tables

**Table 1:** Sociodemographic characteristics of MDR-TB patients in SPSH, 2020 (*n* = 484).

Variables		Frequency	Percent
Age at diagnosis (years)	≤24	125	25.8
	25–30	131	27.1
	31–40	127	26.2
	≥41	101	20.9
	Median age (IQR)	30 (24–39)	—

Sex	Male	236	48.8
	Female	248	51.2

Region	Addis Ababa	297	61.4
	Oromia	120	24.8
	Amhara	38	7.9
	Tigray	9	1.9
	Somali	9	1.9
	SNNPR	9	1.9
	Afar	2	0.4

Residency	Urban	401	82.9
	Rural	83	17.1

Occupation	Government employee	185	38.2
	Student	98	20.2
	Housewife	59	12.2
	Farmer	57	11.8
	Merchant	53	11.0
	Self-employed	20	4.1
	Others	32	2.5

**Table 2:** Clinical characteristics of MDR-TB patients in SPSH, 2020 (*n* = 484).

Variables		Frequency	Percent (%)
Methods of diagnosis	Gene Xpert	359	74.2
	Culture	72	14.9
	Line probe assay	46	9.5
	Clinically suspected	7	1.4

Type of MDR-TB	Pulmonary TB	389	80.4
	Extra-pulmonary TB	95	19.6

Initial sputum smear result (*n* = 389)	Positive	87	22.4
	Negative	302	77.6

Previous treatment for TB	Yes	329	68.0
	No	155	32.0

Baseline weight	<43 kg	172	35.5
	≥43 kg	312	64.5
	Median (IQR)	45.5 (40–52)	—

Substance use	Nonusers	363	75
	Alcohol	105	21.7
	Khat	14	2.9
	Khat and alcohol	1	0.2
	Smoker	1	0.2

Number of drug used for MDR-TB therapy	4	117	24.2
	5	363	75.0
	≥6	4	0.8

Initiation of MDR-TB medication	At the day of diagnosis	392	81.0
	Within 10 days of diagnosis	91	18.8
	After 30 days of diagnosis	1	0.2

Drug adverse effects	Yes	463	95.7
	No	21	4.3

Type of adverse effects (*n* = 463)	GI symptoms	332	68.6
	Peripheral neuropathy	139	28.7
	Hypothyroidism	25	5.2
	Ototoxicity	31	6.4
	Psychiatric symptoms	33	6.8
	Hypokalemia	118	24.4
	Drug-induced hepatitis	44	9.1

Clinical complication	Yes	96	19.8
	No	388	80.2

Type of complication	Pneumonia	59	61.5
	Hemoptysis	16	16.7
	Pneumothorax	13	13.5
	Arthritis	7	7.3
	Cor pulmonale	1	1.0

Presence of lung cavities (*n* = 389)	Yes	57	14.7
	No	332	85.3

IQR: interquartile range.

**Table 3:** Comorbidities among MDR-TB patients in SPSH, 2020 (*n* = 484).

Variables		Frequency	Percent (%)
HIV coinfection	Yes	96	19.8
	No	388	80.2

ART regimen (*n* = 95)	TDF-3TC-EFV	92	96.8
	AZT-3TC-EFV	3	3.2

CD4 count (*n* = 96)	Median CD4 count in cells/mm^3^ (IQR)	163.5 (129.75,197.75)	

Anemia	Yes	133	27.5
	No	351	72.5
	Median (IQR)	13.4 (12.2, 14.5)	

Comorbidities (other than HIV)	Yes	49	10.1
	No	435	89.9

Types of comorbidities (other than HIV) (*n* = 49)	Diabetes	19	38.8
	Cardiac disease	18	36.8
	Hypertension	8	16.3
	Asthma	3	6.1
	Renal disease	1	2.0

TDF-3TC-EFV: tenofovir disoproxil fumarate-lamivudine-efavirenz, AZT-3TC-EFV: azidothymidine-lamivudine-efavirenz, IQR: interquartile range.

**Table 4:** Treatment outcome of MDR-TB patients in SPSH, 2020.

Variables	Treatment outcome	Frequency	Percent	95% CI
MDR-TB treatment outcome	Cured	315	65.1	61.0, 69.4
	Completed	125	25.8	21.9, 30.0
	Successful treatment outcome	440	90.9	88.2, 93.4
	Lost follow-up	20	4.1	2.5, 6.0
	Death	24	5.0	3.1, 7.0

Survival outcome	Censored (0)	460	95.0	93.0, 96.9
	Event (1)	24	5.0	3.1, 7.0

**Table 5:** Cox proportional hazard regression analysis of predictors for time to death outcome among 484 MDR-TB patients in St. Peter's Specialized Hospital, 2020.

Covariates	Event^d^	Censored^o^	Crude HR	Adjusted HR
Sex				
Male	8	228	1.00	1.00
Female	16	232	1.94 (0.83, 4.53)^	1.56 (0.62, 3.93)

Baseline weight				
<43 kg	14	158	2.6 (1.14, 5.24)^*∗*^	0.79 (0.32, 1.94)
≥43 kg	10	302	1.00	—

Baseline hemoglobin				
Anemic	17	116	6.74 (2.8, 16.3)^*∗∗∗*^	**4.26 (1.65, 11.02)**
Not anemic	7	344	1.00	1.00

Previous treatment for TB				
Yes	10	319	1.00	1.00
No	14	141	3.1 (1.37, 6.95)^*∗∗*^	1.74 (0.31, 1.80)

Clinical complication				
Yes	9	87	2.4 (1.05, 5.5)^*∗*^	**2.55 (1.06, 6.10)**
No	15	373	1.00	1.00

HIV status				
Positive	16	80	8.65 (3.7, 20.2)^*∗∗∗*^	**5.7 (2.2, 14.51)**
Negative	8	380	1.00	1.00

Presence of lung cavities				
Yes	5	52	2 (0.75, 5.4) ^	2.52 (0.90, 7.02)
No	19	408	1.00	—

^d^Event in this study was death; ^o^censored include cured, completed, and lost follow-up. ^*∗∗∗*^= *p* < 0.001, ^*∗∗*^= *p* < 0.01, ^*∗*^= *p* < 0.05, ^ = *p* < 0.25, HR = hazard ratio.

## Data Availability

The data used to support the findings of this study are available from the corresponding author upon request.
